# Mothers' accounts of mealtime and feeding challenges for children with Tourette syndrome or persistent tic disorders

**DOI:** 10.3389/fpsyt.2022.936796

**Published:** 2022-08-01

**Authors:** Sandra-Eve Bamigbade, Samantha L. Rogers, Wendy Wills, Amanda K. Ludlow

**Affiliations:** ^1^Centre for Research in Public Health and Community Care, University of Hertfordshire, Hatfield, United Kingdom; ^2^NIHR Applied Research Collaboration, Cambridge, United Kingdom; ^3^Department of Psychology and Sports Sciences, University of Hertfordshire, Hatfield, United Kingdom

**Keywords:** mealtimes, Tourette syndrome, tic disorders, sensory sensitivity, eating behaviour

## Abstract

Parenting a young person with a tic disorder can present daily challenges to families struggling to manage their child's tics and establish routines. Research recognises that tics can be problematic to everyday activities, however no attention has been given to mealtimes, arguably an important family activity closely related to quality of life of the family. The current qualitative study aimed to investigate the mealtime experiences of families with a child with a tic disorder from the perspective of mothers, looking at mealtime challenges, their impact and how these challenges are navigated. Seventeen mothers with children diagnosed with Tourette Syndrome (TS) or a Persistent Tic Disorder (PTD) (aged 3–14) took part in semi-structured interviews. Interpretative phenomenological analysis of 17 semi-structured interviews resulted in seven subthemes which were grouped under two superordinate themes: (1) tics as a barrier to positive mealtime experiences and (2) eating behaviours and other mealtime challenges. The findings highlight tics to create functional mealtime challenges, affecting a young person's ability to eat, drink and be seated, with mothers noting the family dynamic was often intensified and compounded by additional challenges related to their child's tics and comorbidities. Tics also have the power to disrupt the conviviality of mealtimes. For example, eating out-of-home can be especially challenging, with restaurants being high-pressure environments for young people with tics and their families. The cumulative effect of dissatisfaction, stress and additional foodwork can have a diminishing effect on maternal and familial resilience and wellbeing. Mealtime-related interventions need to be considered to help increase confidence and skills in managing mealtimes.

## Introduction

Tourette syndrome (TS) is a neurodevelopmental disorder characterised by motor and vocal tics ranging in form, frequency, complexity, and intensity (1). Tourette Syndrome differs from Chronic Motor or Vocal Tic Disorder and Provisional Tic Disorder in two ways: the type of tic and its persistence. For a Chronic Motor or Vocal Tic Disorder diagnosis, an individual only needs to have the presence of a vocal or motor tic for over a year to be eligible for diagnosis; for TS, the individual would require both types of tics for over a year to be eligible for diagnosis. A Provisional Tic Disorder diagnosis would apply when tics have been present for less than a year. In addition to the core characteristics, there is research drawing attention to feeding challenges in children with TS (2–4); however, these studies have yet to adequately capture the impact of feeding difficulties on family mealtimes.

Mealtimes have been described as the cornerstone of family life, with there being no other daily activity that families share together with such regularity, with around 72.8% of school-aged children found to eat dinner with at least one parent every night of the week (5). Mealtimes also provide families an opportunity for a daily structured routine, often supporting larger family goals as well as communication (6). Larson and colleagues (7) have also suggested that family meals are a symbol of family unity. However, while family mealtimes can be a source of joy, they can also be a source of stress and dissatisfaction as mothers struggle to recreate their ideal family mealtimes (8).

Mealtimes can be a general source of tension, yet in families with a selective eater, this stress is magnified [for review, see (9)]. For example, selective eating, also known as fussy eating, whereby children reject a high proportion of familiar and novel food, is common during early childhood (10). However, food selectivity has been shown to be more persistent and severe in children with TS compared to their typically developing peers, presenting beyond the normal developmental stage of 6 years of age (3), with food avoidant behaviours such as food selectivity, food neophobia and restrictive eating remaining into adulthood (4). Moreover, children with TS have been found to show similar levels of food fussiness compared to other neurodiverse children including Autism Spectrum Disorder (ASD) and Attention Deficit Hyperactivity Disorder (ADHD) (2), even when accounting for levels of comorbidity.

Research addressing the mealtime experiences in neurodiverse families has largely focused on families with a child with ASD, with mealtime barriers revolving around child's selective eating and mealtime behaviours (11–14). For example, a Canadian study by Rogers et al. (15) found that mothers of children with ASD (aged 4 to 11 years) had to contend with sensory aversions, a need for sameness, rigidity, and food jags (repeatedly eating the same meal for an extended period before cycling to another safe meal). Mothers also described their child as engaging in disruptive mealtime behaviours such as constantly getting up from the table, fidgeting, food refusal and attention seeking during mealtimes (16, 17).

Ausderau and Juarez (18) also found mothers described mealtimes as unsatisfactory due to their child's selective eating, disruptive mealtime behaviour and the additional labour they had to undertake to make mealtimes “work” for their families. For example, mothers had to create individualised mealtime routines to accommodate the needs of their child with ASD, however, adaptations came at a cost to other members of the family. Namely, typically developing siblings who needed to be mother's “little helper” and model “good” behaviours, and mothers who had to undertake additional foodwork with little support or understanding from their partners, friends, and relatives. Consequently, mothers often expressed a sense of hopelessness and dissatisfaction at being unable to create the mealtime experiences they desired due to the eating and mealtime behaviour of their child with ASD (17).

The ASD literature highlights the complexity of maternal mealtime stress and may provide some insight in the experiences of TS due to shared traits, characteristics, and comorbidity (19). Important questions remain regarding how mothers of children with TS, navigate mealtimes and what personal costs are associated with adaptations and additional foodwork. This is particularly important to address, given the suggestion that families of children with TS struggle with daily routines and will often change the timing of meals to accommodate their child's tics (20).

To our knowledge this was the first qualitative study addressing mothers of children with tics experiences of mealtimes. Mothers were chosen, not only to be synonymous with the existing ASD literature, but also because mothers typically tend to undertake most domestic foodwork (21); namely meal planning, cooking, and cleaning and often the emotional toll of labour associated with foodwork and feeding (22). Even during the COVID-19 pandemic, when two parents were present within the family home, mothers spent more time than fathers doing foodwork (23).

## Materials and methods

We aimed to understand mealtimes in families with a young child with a tic disorder from a maternal perspective. For this research, a meal was considered a family meal if at least one adult and one child were seated for a meal together, even if one of them was not eating (24). For data collection and analysis, we referred to interpretative phenomenological analysis (IPA) guidelines by Smith et al. (25). This choice is congruent with our aim to uncover the meaning given by these mothers to their experiences (26).

### Participants

Seventeen white British mothers with children diagnosed with TS (*N* = 15) or a PTD (*N* = 2) took part. Almost all the participants were reported to be diagnosed with more than one comorbidity and or awaiting further diagnoses (see Table 1). The comorbidities reported were as follows: OCD (*n* = 9), Anxiety Disorder (*n* = 9), ADHD (*n* = 8), Sensory Processing Disorder (SPD, *n* = 5), Learning disability (*n* = 5), and ASD (*n* = 3). Several mothers also reported that their child was awaiting further diagnoses including ASD (*n* = 4), ADHD (*n* = 1) and anxiety (*n* = 1). Mothers also reported the following traits in their children that were not diagnosed nor awaiting diagnoses: SPD (*n* = 5), OCD (*n* = 1), and ASD (*n* = 2). This sample is thought to reflect the spectrum of presentations within this population; with TS being a multifaceted condition with complex clinical presentation due to high comorbidity rates. Mothers were predominantly employed, either full-time (*N* = 7) or part-time (*n* = 4), and most mothers lived with their partner (*N* = 16) and had other children living in the household (*n* = 13). Four mothers also reported that someone else in their household had a tic disorder.

**Table 1:** Parent, child, and family characteristics.

**Participant characteristics**			**Child characteristics**				**Household characteristics**	
**Pseudonym**	**Mother's paid employment status**	**Partner's paid employment status**	**Pseudonym**	**Age**	**Diagnoses^a^**	**Medication^a^**	**Immediate relative with TS/PTD**	**Other children in the home**
Amy	Full-time employment	Full-time employment	Talia	13yo	TS plus 2 NDD	None	No	No
Caroline	Not in paid employment	Full-time employment	Adam	3yo	PTD plus 1 LD	None	Partner with TS, youngest suspected PTD	Yes
Charlotte	Not in paid employment	Not in paid employment	Thomas	14yo	TS plus 2 NDD and 1 MHD	Takes antidepressant, antipsychotic, and antihistamine	No	Yes
Ciara	Not in paid employment	Full-time employment	Justin	11yo	TS plus 1 NDD and 1 trait	None	No	Yes
Harriet	Not in paid employment	Full-time employment	Max	8yo	TS plus 1 NDD and 1 trait	None	No	Yes
Jackie	Full-time employment	Part-time employment	Ivy	14yo	TS plus 1 MHD, 1 NDD and awaiting 1 more NDD	None	No	No
Jessica	Part-time employment	Full-time employment	Warren	11yo	TS plus 2 NDD and 1 MHD	Takes antidepressant and melatonin	No	Yes
Lauren	Full-time employment	Full-time employment	Finley	13yo	TS plus 1 NDD and awaiting 1 more NDD diagnosis	Takes stimulant	No	Yes
Marisa	Full-time employment	Full-time employment	Lottie	4yo	PTD	None	Has TS	No
Naomi	Not in paid employment	Full-time employment	Oscar	8yo	TS plus 1 NDD, 2 MHD and 1 LD	None	No	Yes
Polly	Full-time employment	N/A^b^	Zack	14yo	TS plus 1 NDD and 1 MHD	None	No	Yes
Rebecca	Part-Time Employment	Full-time employment	Ryan	13yo	TS plus 1 NDD, 1 MHD and awaiting 2 more NDD diagnoses	Takes antidepressant	No	Yes
Rita	Not in paid employment	Full-time employment	Effy	13yo	TS plus 3 NDD and 1 trait	Takes alpha-agonist hypotensive agent and melatonin	No	Yes
Serena	Part-time employment	Full-time employment	Felix	11yo	TS plus 2 NDD, 1 MHD and 2 traits	Takes stimulant antipsychotic, antidepressant, antidiuretic, and melatonin	Younger child, possible PTD	Yes
Sophie	Part-time employment	Full-time employment	Jack	10yo	TS plus 1 NDD, 1 LD, awaiting 1 NDD and 1 MHD diagnosis and has 1 trait	Takes lpha_2A_-adrenergic receptor agonist	No	Yes
Susan	Full-time employment	Full-time employment	Annabelle	13yo	TS plus 3 NDD, 1MHD and 3 LD	Takes antipsychotic	No	Yes
Yasmin	Full-time employment	Full-time employment	Isaac	12yo	TS plus 2 NDD, 1 MHD and 1 trait	None	Partner has tics	No

a *The specific diagnoses of YP and list of medications that they take have not been listed within the table in order to preserve confidentiality*.

b *Polly's ex-husband lives separately, so no other caregiver was living in the family household*.

*LD, learning disability/disabilities; N/A, not applicable; yo, years old; NDD, Neurodevelopmental diagnosis/diagnoses; MHD, mental health diagnosis/diagnoses*.

*Columns two and three focus on paid employment status; therefore, those who work within the family home as homemakers and carers are classified as “not in paid employment.” This does not serve to discredit the value of their invisible domestic labour and is only used to provide context for caregiver work patterns and to classify whether a family is a single- or dual-earner household as both are important factors worth considering when exploring family mealtimes (27, 28)*.

Mothers were recruited through a short online advert disseminated *via* Tourette's Action, Tourettes Hero and private Facebook groups which support families with children with TS. Social media channels such as Twitter and Redditt were also used to aid recruitment. Aligning with the principles of IPA, the relevance of findings is dependent on the richness of the narratives as opposed to the sample size (29, 30). We deliberately chose a larger pool of participants than is usually included when using IPA (typically between *N* = 3–15), to explore the breath of mothers' individual experiences but also reflecting the heterogeneity in the symptoms and comorbidity of children presenting with a tic disorder. Mothers who were interested in the study were advised to contact the lead researcher for more information and were then sent an information sheet that detailed the study's aims and objectives and how data would be used and protected. Once a date, time, and location (virtual or in-person) were agreed upon, mothers were sent an overview of the interview schedule to know what type of questions to expect. All participants provided written and verbal consent and were assured of their anonymity and right to withdraw at any stage. Participants also provided consent for their interview to be recorded for transcription purposes. Ethical approval for this research was obtained from the University of Hertfordshire University Ethical Advisory Committee Protocol Number: aHSK/PGT/UH/03340(5) and the research was performed in accordance with the Declaration of Helsinki.

### Data collection

Empirical literature for assessing mealtime challenges in children with neurodevelopmental disorders guided the creation of the interview schedule. The schedule was further verified by a parent of child with TS (not included in the study) alongside members of the research team. The first part of the schedule captured contextual information about participants, their child, and their household. Notably, parental occupation and work pattern; target child's age, sex, and diagnosis; and family structure. The second part of the schedule focused more specifically on mealtimes, asking the following:

- When was the last time you sat down to eat a meal with your family? Can you describe that mealtime for me?
- What types of food and drink does your child like or dislike?
- How, if at all, does your child's Tourette/tics influence your mealtime experiences or their eating behaviour?
- Does your child take any medication? If so, have you noticed any changes to their appetite and weight? If yes, can you talk to me about that?
- When was the last time you ate out as a family? Can you describe it to me?
- Do you have any future concerns about your child's mealtimes?

Interviews were conducted by the lead researcher between October 2018 and August 2020, with only two participants, Jackie and Susan, interviewed during the COVID-19 pandemic. Fourteen interviews were conducted virtually, using an online platform such as Zoom; the remaining three interviews were held face-to-face at participants' home at their request (pre-COVID-19 pandemic). Interviews lasted from 49–182 min. All interviews were recorded for transcription purposes and transcribed verbatim by the researcher.

### Data analysis

The first author read each transcript multiple times, before the data were analysed. In accordance with quality guidelines for IPA, reflexive conversations occurred amongst the four members of the research team (31). For each transcript, emergent themes and interpretations were noted alongside divergent and convergent themes to highlight the unique experience of each participant (31). To ensure further credibility, triangulation occurred through consultations with the research team, and feedback was also sought from participants of the study, with no changes requested.

## Results

Analysis resulted in five subthemes grouped under two superordinate themes: (1) Tics as a barrier to positive mealtime experiences, and (2) eating behaviours and mealtime challenges, see Table 2. These themes captured mothers' thoughts and feelings surrounding their family mealtime experiences and their child's eating behaviours. Some of the words mothers used to describe mealtimes were stressful, uncomfortable, chaotic, messy, and fragmented. Each theme articulates these descriptors more fully while situating them within the context of distinct behaviours and characteristics associated with tic disorders and comorbidities.

**Table 2:** Theme structure for mothers of young people with TS.

**Superordinate themes**	**Subthemes**
Tics as a barrier to positive	Functional challenges
mealtime experiences	Disruptive tics and fragmented mealtimes
	Self-consciousness and anxiety when dining out
Eating behaviours and	Food preferences and feeding practises
mealtime challenges	Conflicting mealtime expectations

### Tics as a barrier to positive mealtime experiences

Mothers described their child experiencing an assortment of tics, all of which were portrayed to have varying effects on mealtimes. This superordinate theme consists of three subthemes: (1) functional challenges, (2) disruptive tics and fragmented mealtimes, and (3) self-consciousness and anxiety when dining out.

### Functional challenges

On a functional level, mothers reported that tics impaired their child's ability to eat and drink uninterrupted. In most instances, these functional challenges were more impactful on their child's behaviour than the mealtime experience. For example, Jackie noted that their daughter's head and neck tics made it hard for them to eat “*because of the neck jerking, it'll interrupt her from her eating pattern.”* Whereas oral tics were noted by Amy and Polly:

“When, when she was doing the lip rolling umm, sometimes she would find it difficult eating and the jaw slamming. Sometimes like she'll bite her tongue or the inside of her cheek.” (Amy)

“He had one for a while that was like (demonstrates mouth wide open and eyes closed tic) like this, opening his mouth. But he still ate. It was just that he would chew his food and then (mouth open tic) in between.” (Polly)

Jessica described how Warren's throat tic sometimes made it difficult for him to finish his meal and left him “*panicked*.”

“He's choked before because umm… it went to the back of his throat, and he tried to clear his throat, but it got stuck so he choked. It scared him a bit. But then because he panicked, his tic heightened so he was doing it constantly so he couldn't eat. He's done that quite a few times.” (Jessica)

Others highted how tics usually interacted with what would be considered good table etiquette. For example, Amy related her daughter's tic spillages to her limb tics whereas, in the case of Yasmin, the issue related to her son's distractibility during mealtimes.

“I have to feed him, not because he's incapable of feeding himself, but because he'll just sit there and be distracted, maybe because he's thinking if he puts the fork in his mouth, he'll, he'll tic.” (Yasmin)

### Disruptive tics and fragmented mealtimes

Tics were also described as being disruptive to family mealtimes, although the disruption depended on type of tic and its severity. For example, tic severity was cited as disrupting mealtimes in several ways. One was delaying mealtimes until tics waned whereas the another was the perpetual movement during meals, as children struggled to sit still.

Importantly a few mothers described their child's tics as influencing the timing of their family meals, as children could not sit down for dinner shortly after returning from school due to what mothers perceived to be “tic rebounds.” Mothers rationalised that it was more effective to delay dinner than to try and force their child to sit at the table.

“He'll hold them in and try and suppress them as much as he can […] but eventually when he gets home, it's like taking a lid off a pressure cooker, and all of those tics have to get out. So, at the time he's coming home, umm when you think actually, we should be sitting down and we should be having dinner, uh we can't do that because he needs at least 2-h just to go into his room, have that space on his own, not really have any interaction.” (Lauren)

While mothers, such as Lauren, were able to accommodate an increase in tic severity by pushing mealtimes back, this only resolved the challenge of getting their child seated at the table. Many mothers also noted that it was a struggle to then keep them there.

“It's just utter chaos, he don't sit down at the table, he walks around, he gets upset… uhhh… I don't know. And then everybody gets stressed.” (Jessica)

Mothers described being acutely aware of their child's need for movement and often came to understand that movement was a necessity for their child that should not, and could not, be policed. As such, mothers often made concessions for their child, allowing them to move around as needed, but maintained an expectation that their other children stay seated throughout the meal.

“Like, he's always found it hard to sit still, and he's never been able to sit at the table, but I sort of knew that as a mum just let him bounce around a lot if he needed to.” (Ciara)

“He wants to move around […]…it tends to be 3 of us sitting at the table with Oscar bobbing about. Umm… and… I guess… it's sort of the things that goes with the Tourette's I suppose.” (Naomi)

Rebecca described her family mealtimes as being negatively impacted by Ryan's spitting tic, explaining it was particularly challenging for her younger son with ASD, Josh, to ignore Ryan's tics.

“Josh's got a lot of anxiety around sitting at the table where he's likely to be spat at […] he feels sometimes not safe at the table, he didn't feel like he was comfortable eating, so we kind of made a decision that uh he was better off eating and not associating fear with food and not eating at all.” (Rebecca)

Rebecca explained that the only way she was able meet her sons' varying needs was to have both eating in separate parts of the house. Rebecca willingly scarified the family meal in favour of her children's long-term wellbeing and future mealtime enjoyment, stating: “*I just hope that one day they'll come back to the table, and we can eat together because they are not anxious about food*.”

This fragmentation of the family meal was also noted by Marisa, but this time it was her daughter Lottie with PTD who ate alone, separate to the rest of the family unit. Marisa explained that she preferred Lottie to eat alone as this shielded Lottie from being reprimanded by her dad for her expulsive tic which drives her dad “nuts.” This allowed Lottie to tic freely without feeling “*like she's bothering her dad*.”

Expulsive tics such as *stabbing, hitting, and kicking* tics, were frequently described by the mothers as not only disruptive but also harmful to others. At times, mothers described these tics as being painful and having a negative effect on enjoyment of mealtimes. For example, Ciara described herself as being “*traumatised*” by Justin's tics making it difficult for her to enjoy mealtimes.

“Just this week, again, I've been starting to get kicked under the table and having to stop that because you just don't want that when you're eating. […] It's, it's hard to say how difficult that is. You know, I think I've actually been quite traumatised over the years from the amount of being jumped on and touched and umm I say kicked, but it's not aggressive, it's just overly boisterous […].” (Ciara)

While Ciara understood that Justin did not intentionally want to hurt her, she nonetheless felt unsafe. For example, Ciara explained that she felt one of the reasons why she “*got ill*” was due to “*the constant bracing yourself because you never know when you're going to be bundled into*.” Fortunately for Susan, her table was able to maintain distance between her and Annabelle, which meant that being hit during mealtimes was no longer a challenge.

“We've got enough space. We're lucky enough to have six seats at the table. So, we leave a gap in the middle. I used to sit next to Annabelle, but I got stabbed and hit. One mealtime, I got hit on the head with a spoon over 30 times. And it really does hurt.” (Susan)

Susan demonstrated her dedication to persevere through the mealtime, being hit and hurt “*over 30 times*”, highlighting not only the impact of the tics on others (e.g., “*and it really does hurt*”) but the lengths taken by mothers to ensure everyone's needs were considered and met. Moreover, changes were made to accommodate tics at mealtimes as opposed to centring attention on tics and their impact.

### Self-consciousness and anxiety when dining out

Many reported their child's desire not to have attention drawn to them would influence every aspect of dining-out, from the frequency of dining out, to the location and even the time of eating, causing stress for all members of the family.

“[…] eating out at a restaurant, depending on his mood and where, what his tics are like can vary massively […] some days it's literally like having a bull in a china shop. Trying to get him to sit down, sit still, he's ticking, not throwing his salad bowl across the table umm… but we try to avoid those places to be honest because it's not nice for anyone.” (Serena)

Mothers whose children were not overwhelmed by noisy environments tended to opt for child-friendly establishments where their child could assimilate by blending into the background.

“We go to a family place, you know like Carveries and things like that because they're darker, they're loud anyway, they're busy, so you just blend in.” (Serena)

Other mothers preferred to request quiet tables and inform the staff and fellow diners of their child's condition. This was perceived by mothers to help to ease their child's anxiety and minimise staring. Mothers also reported seating preferences. For example, Amy explained that Talia “*she just prefers to be in the corner*” to be less conscious of onlookers.

“We have to book in advance and ask for special tables, and then all the waiters have to know. Annabelle likes me to tell everybody and people on the tables around us. It makes her feel more comfortable that they have some understanding.” (Susan)

In addition to controlling the environment to create less pressurised experiences, mothers also noted that their child would try to suppress their tics. The challenge with this approach was the abrupt end to mealtimes when their child was no longer able to cope and suppress tics. Sometime this resulted in families being unable to go out for a meal if their child had a bad tic day.

“[…] there will be times where he will say ‘mummy can we go home now?' or you know ‘I'm getting a headache' or umm he'll say or ‘I've got a tummy ache' and that I know that he can't, he needs to release it. And if we're halfway through the meal then I'll say to him ‘come on, do you want to come with me to the toilet' and him and me will go off separately, and then he'll just be able to do his own little thing. Tic away and no one else is watching him, and then he feels comfortable to go back to the table.” (Sophie)

Charlotte and Lauren were the only mothers whose sons refused to dine out with their family as they were now old enough to decide to stay home. While Lauren and Charlotte appreciated that it was easier, they often felt uncomfortable about leaving them alone and were concerned about their sons' social withdrawal.

“It's difficult to eat out because he doesn't like attention being brought to him. And umm he'll wear a hoodie and have it over his head umm because that's some type of protection for him that, you know, he's kind of hiding behind. If we do go out, we don't tend to take Finley with us. And he's 13, and he can make that decision. It is not enjoyable for him, which is really/ it's a shame.” (Lauren)

“In fact, I can't think of the last time that [he] came out with us for something to eat.” (Charlotte)

### Eating behaviours and mealtime challenges

This superordinate theme discusses how mothers viewed their child's eating behaviours and the role sensory sensitivity and rigidity played in making mealtimes stressful and conflictual. This superordinate theme consists of two subthemes: (1) food preferences and feeding practises and (2) conflicting mealtime expectations.

### Food preferences and feeding practises

Several mothers described their child's food preferences as a source of stress, as they felt that their child's food preferences were limited, albeit to varying degrees. Mothers who described their child as a selective eater or having pronounced food preferences tended to attribute their child's dietary range to sensory aversions.

“He seems to have heightened sense of smell, like he finds certain textures really uncomfortable umm and then he just/ he just tastes things, he only likes really bland things.” (Harriet)

“She'll say if it smells wrong or looks wrong, it feels wrong, and there's like an invisible force field, and she just can't do it.” (Rita)

Mothers often described instances where it became apparent to them that their child was genuinely struggling with sensory properties and that their refusal was more than merely behavioural.

“For instance, and he's a good boy, and he tries his hardest, but he tried to eat a piece of sweetcorn, and it took him 15 min. And it was 15 min of crying, you know, at the noise in his ear of crunching it.” (Harriet)

Over time, mothers accepted that controlling feeding practises were counterproductive and appeared to feel powerless and defeated. Mothers reported feeling pressurised as their child's meal had to be served in a particular way, most commonly with each meal component separate on the plate.

“Beans can't touch his food […] so jacket potato and beans, umm they have to go in a cup […] (Serena)

“[…] he doesn't really like bean juice. So, you have to drain the bean juice up the beans so it's not as wet. And he likes the beans separate to the chips.” (Lauren)

Rather than feeling defeated and helpless at changing their child's diets, Serena stressed the importance of knowing what was realistically achievable and working with her son's preferences when increasing acceptance of otherwise refused foods.

“[…] he hates things with two textures. Like you cannot give him yoghourt with fruit in. Or bits in, that's a no, no. […] I learnt from a very young age when he was little that that's just not something I'm going to force him to have.” (Serena)

Mothers who did not feel this burden were less likely to perceive their child's dietary preferences as a challenge and were less likely to encounter mealtime battles. For example, Marisa and Caroline both described their children with PTD as selective eaters, yet this did not appear to be a challenge nor source of stress; seemingly because they both were able to alleviate concerns about nutritional deficiencies.

“[…] she eats breakfast and lunch and snacks at school so… uhh she/ I know that she's having very varied meals there […] so I'm not going to worry about her too much about what she's eating for dinner.” (Marisa)

Interestingly, a couple of mothers also noted that the burden to nourish their child felt heavier due to their child's diagnosis.

“[…] when your child has a chronic condition, and there's no cure and… there's precious little help from the health service, you have to work it out for yourself […] I am giving him as healthy a meal as possible, and I hope that is at least helping things not get worse.” (Ciara)

Ciara's desire for Justin to have a healthier diet than he would like often led to mealtime conflict, stating that “*it just feels like a battle all the time.”* In the end, mothers often described themselves as feeding their children their preferred foods, so to avoid them missing a meal. For example, Harriet described having to find a balance between “*starving your child”* and making sure they are “*getting proper nourishment”* as being “*extremely stressful.”* Even when mothers tried their best to accommodate their child's preferences, they could not always ensure their child would eat the meal as some children's preferences were unpredictable. This was disheartening for mothers like Jackie, who felt that even despite their best efforts to make a meal their child would enjoy, they were still unable to “*get it right*.”

“It can be a bit disheartening after you've spent an hour or more cooking and then [she] doesn't like that, can't eat it. And I couldn't have predicted that outcome.” (Jackie)

The levels of accommodation for food varied, as did the impact of this additional labour on mothers' stress levels. A few mothers prepared separate meals for their child. Although, in the case of Lauren, she prepared individual meals for the whole family due to lack of taste synchronicity. Lauren likened her household to a “*café where everyone has a different meal.”* While she first cited this as a source of stress, she later recanted and explained that while it “*sounds like it would be stress city […] it does become the norm.”* While Lauren had acclimatised to making several meals, the idea of cooking multiple meals was stressful for others. In such cases, mothers opted for meals that could easily be modified to meet everyone's needs.

“[…] say I was doing a chana masala or something, a chickpea curry, Max would have the chickpeas and the rice but no sauce so it's not really our dinner at all, but that's, that's what he'd eat.” (Harriet)

“I give them an option, and we try and come at one we all agree at because I was cooking different meals for everybody. […] I'll do something where Annabelle could have say, chicken in a wrap and Ella will eat a Caesar salad.” (Susan)

### Conflicting mealtime expectations

Expectations surrounding family mealtimes appeared to be a notable factor influencing how satisfied mothers were with their family mealtime experiences. Mothers noted two main conflicts, conflict within themselves between what they want and what their reality was, and conflict between their expectations and that of their partners. For example, Caroline held onto an expectation that her family mealtimes could improve but also recognised that despite all her best efforts thus far, mealtimes were still “*crazy.”* Both mothers held strongly onto their expectations, although in Caroline's case, her “*micromanaging”* of mealtimes was described as a source of stress for her family.

“[…] we used to have them as kids, it should be like a social time where everyone is happy, and you're catching up with the day or/ but it's not because Oscar will want to get up or ‘that's not right,' ‘that's not right.' I think, maybe I sort of sit there and think, ‘oh, they're gonna'/ oh I don't know, not like the Waltons but you know be like ‘this is lovely, you've worked so hard, this is delicious' (laughs). But it rarely ever is […] it's like a battleground really to sit down as a family […].” (Naomi)

“Like the number of times that we've been successful at that is so rare that that's really creating stress for my family because I just keep plugging away at it. Like I keep expecting that we'll be able to […] every day, all day, like our lives revolve around the kitchen. That we're making food, we're cleaning food, we're eating food, like they're just like so over it.” (Caroline)

Naomi's quote captured the discrepancy between what she felt mealtimes should be, a wholesome family activity, vs. what they were, a “*battleground.” “it's not like relaxing, we all sit there you know… it's quite tiring.”*

Naomi commented that even when Oscar was a baby, he refused homemade baby food which meant she could not be the “*smug mummy”* she wanted to be. Her motivation to undertake extensive foodwork appeared to be embedded in her desire to derive joy from the pleasure her family experienced when they ate her meals. Similarly, other mothers noted this challenge as they felt their foodwork was not enjoyed, nor appreciated, as they had hoped.

“I think it's, it's a challenge trying to predict sometimes whether she's going to like what I'm cooking. That can be frustrating, and that could become a challenge if I allowed it. […]it can be a bit disheartening after you've spent an hour or more cooking and then doesn't like that, can't eat it. And I couldn't have predicted that outcome.” (Jackie)

“I want food to be joyful. I want it to be something that can be social, and I [can't] figure out how to do that when other people won't cooperate (laughs).” (Caroline)

For Harriet, neither she nor her family were able to derive joy from the meal that she had tirelessly prepared, with Max's food refusal and “*meltdowns*” created a stressful mealtime atmosphere.

“[…] there has been times when I have just picked up my plate because I've had a knot in my stomach from the screaming, picked up my plate and had to go to a different room to eat my meal because I might have made something that took me an hour, an hour and a half, and I can't even taste it because my child is screaming because the smell from his plate or even having to do it. Umm, so it can be very stressful.” (Harriet)

Notably, maternal identity was heavily tied to what their child ate and as such, it was challenging for mothers to let go of mealtime expectations entirely. Jackie captured this sentiment as she expressed guilt and disappointment tied to Ivy's eating behaviour.

“I mean, for me as a mum, I have to not be too disappointed if, you know, I can spend quite a lot of time cooking and preparing and think it's going to be fine. And then if she says, ‘I can't eat it,' I've then got the guilt of ‘well do I have to go back into the kitchen and cook another meal?” (Jackie)

The very few mothers who accepted that they had no control and released all expectations about mealtimes appeared to be the most content. Rebecca and Lauren captured this best. Rebecca accepted her fragmented mealtimes, while Lauren accepted the need for multiple meals.

“It would be nice to just cook one meal, and everybody eat it [but] we're not that family. So, you've got to adapt.” (Lauren)

Rebecca also recognised that while she “*would like everyone to be in the same place”* that this simply was not possible due to her sons' conflicting needs. For Rebecca and Lauren, mealtimes were simply for getting everyone fed.

Another challenge mothers noted was between their expectations and those of their partners. In most cases, mothers reported their partner to be stricter or less understanding than they were. Both expectations and parenting style were noted to have intergenerational influences. In the example below, Jessica described why she believed she was stricter than her husband, Jim.

“We were brought up differently. Jim didn't […] sit and eat with his parents, it were always, you know, you … you can sit and eat in there. […] she (Jim's mother) made meals separately for everyone. So, if he didn't want something, he could have something else. Whereas my sort of upbringing were completely different. I, we had a set meal at a set time.” (Jessica)

In cases where mothers believed themselves to be less strict and more understanding than their partners, they also felt the need to advocate on their child's behalf. Like Rita, some of these mothers felt caught in the middle as they empathised with both their partner and their child. Rita articulated this well when discussing her husband's reaction to Effy going out with friends the day after she had a “*meltdown and just absconded and went to the car”* during a family meal.

“He struggles, he struggles with it more than I do. He/ even now so like she had this meltdown in Pizza Hut. I encouraged her the next day, and she went to drama, and he's upset because we rarely go out for family meals or do stuff anymore because of her issues. […] he thought ‘well if she can go out to drama, why can't she go out for a meal with us?,' ‘If she can do what she wants to do, why can't she do what we want to do as a family?.' And how sort of sad it is, and I totally understand where he's coming from because I felt like that in the past, and even now I do.” (Rita)

## Discussion

The study captured maternal perceptions of their family mealtimes, namely the challenges they faced and how they responded to them. Like previous research, barriers to positive family mealtime experiences and sources of maternal mealtime stress tended to focus on selective eating and disruptive mealtime behaviours (8, 32), which were further magnified by the functional and sometimes expulsive nature of the tics. While mothers appeared to understand that sensory sensitivities often underpinned their child's food preferences, they nonetheless desired their child to have a broader diet. When mothers used controlling feeding practises, mealtimes were described to be stressful and conflictual whereas when they were able to see their child as struggling due to sensory sensitivity, it was easier to accommodate their child's food preferences.

Akin to the research in mothers with children with autism, previously described by Suarez et al. (17), the inability to remain seated was cited as being a particular source of annoyance. There was a strong consensus amongst the mothers that tics intensified when they returned home from school, mothers often conceptualised this being a result of tic suppression during the day. However, it is important to note that empirical research does not support a tic rebound effect (33, 34), though these studies only explore rebounds within 40 min of suppression. A plausible reason for this phenomenon could be due to accumulated fatigue and feeling relaxed in their home environment. Regardless of the reason for increased tic severity upon returning from school, the need to delay mealtimes to accommodate the perceived increase in tic severity was noted by several mothers; something which has previously been described in TS (20). While this was disruptive to the family's routine, it was often less disruptive than the presence of certain tics during the meal. For example, mothers depicted an array of disruptive tics including hitting, throwing, and kicking that impacted their child's ability to eat, be seated and stay seated and generally impacted upon other family members' mealtime experiences.

In addition to the practical challenges tics presented, mothers described experiential and emotional challenges. These included affecting the ability of others at the table to relax, and enjoy their meal, as well as self-consciousness when dining-out. Outside of the family home, tics were often an issue, drawing unwanted attention to the family with some families avoiding dining-out regularly. Avoidance of social activities, due to fear of being stared at is a common challenge faced by families with a young person with TS (35, 36), particularly true for socially unacceptable behaviour [e.g., swearing tics (37, 38)]. Families who dined out tended to opt for environments they felt would be more accepting of their child's tics and behaviours; usually family-friendly restaurants where they could blend into the background. While the need for family-friendly environments was also mentioned by mothers of children with ASD, what was deemed suitable varied depending on each child's needs (17). Mothers in the current study preferred louder venues where their child's tics could blend in, whereas mothers in Suarez and colleagues' study required quieter venues to accommodate their child's sensory sensitivity. This finding highlights the varying needs of neurodiverse populations and how environments that might meet the needs of some families may be problematic to others. Finding a suitable environment may be particularly challenging for children presenting with more than one neurodiverse condition (39).

The children's eating behaviours themselves were also noted to be a particular source of mealtime stress. For example, food related challenges included selective eating, food refusal based on sensory sensitivity (taste, texture, and smell) and mealtime behaviour challenges (meltdowns). These eating behaviours were described as creating stressed and strained mealtime interactions, often leading to conflict and additional foodwork. This study supports previous findings highlighting sensory sensitivity to underlie food selectivity in children with TS (2, 3), but reflects the lived experiences and the resulting challenges from these behaviours.

Some mothers frequently used combative language to describe their mealtime interactions with their children, often describing it as a “*battle*.” These mothers tended to be concerned by their child's eating behaviour, which motivated them to assert control over their child's food choices (40, 41). However, mothers often described their attempts to control their child's eating behaviours as leading to a battle of wills, which ultimately ended in a “*meltdown”* and consequently conceding to maintain the peace. The repetition of these experiences led to mothers feeling defeated and exhausted, sentiments echoed about mealtimes by mothers of children with ASD (17, 18, 42). While mothers may think that controlling feeding practises will improve their child's selective eating (41), they may unwittingly further entrench selective eating and create negative associations with food [for review, see (43)].

The current findings also highlight the importance mothers place on family mealtimes and their inability to recreate their desired experiences. Dissatisfaction occurred as result of the incongruency between what mothers desired and their reality, and failure to accept their reality. Mothers in this study who internalised notions of good mothering [e.g., the provision of nutritious home-cooked meals, see (44)], were particularly affected as it challenged their identity. For these mothers, mealtimes appeared to be associated with dissatisfaction with their mealtime experiences, grief for what cannot be, guilt for not being able to recreate the mealtimes they had hoped for, as well as sadness.

The cumulative nature of stressful mealtime experiences and having to accommodate preferences took a toll on mothers; some felt hopeless with no other choice but to give up despite not wanting to, while others surrendered to their reality, opting to give in to keep the peace. For example, Thullen and Bonsall (14) found that disruptive mealtime behaviour, food refusal and mealtime rigidity were all independently associated with increased stress in parents of children with ASD. Mothers of children with a tic disorder may also benefit from interventions to reduce mealtime-related stress, however little support is currently available for these challenges (42, 45). Maternal accounts indicated stress/conflict avoidance as the main overarching goal of mealtimes, which has been shown to be the most common mealtime goal in parents of children aged 1–16 years (46). Therefore, any intervention must be aligned with this goal to be successful. Some practical suggestions to manage mealtime expectations include: providing more food at breakfast or lunch and a smaller meal for dinner; accepting that food will spill, messes will happen, and children will not always be hungry; and allowing more time for meals. However, mealtime interventions are complex mainly as there was not just one profile of mealtime concerns. More research addressing feeding and mealtime challenges is vital, as more knowledge of specific feeding issues for children with a tic disorder may help paediatric therapists plan interventions. These interventions are likely to include various sensory- and behaviour-based techniques (47).

While it has been shown some of the daily challenges mothers and their families faced, this study does not seek to imply that these experiences are representative of all families with a young person with a tic disorder, nor would it be reiterated by the fathers' accounts. For example, this is based on a small sample of self-selected mothers who may have taken part because of their difficult mealtime experiences. Notably, some positive experiences were shared, such as the palpable resilience of these mothers and their commitment to their children and families.

The findings of this study should be considered within the context of its design and limitations. Firstly, this study relied on purpose sampling, which may have biassed the sample towards mothers who place an importance on mealtimes, experience mealtime difficulties and or have children with greater tic severity than those who chose not to participate. The mothers also all identified as White British, thus future research would benefit from a more ethnically diverse sample to explore the intersect of race and culture on mealtime experiences within this clinical population. Thirdly, the children's diagnoses were reported by the mothers, with them being asked to confirm their child had a formal diagnosis. As there was no independent assessment, diagnosis status cannot be confirmed. Relatedly, all but two of the children had a primary TS diagnosis, with two of the youngest children (aged 3 and 4 years) both having a PTD. There is some caution about diagnosing TS and PTD in very young children due to the common transitory nature of tics during this developmental period (48). Nevertheless, in both instances, the children in question had a parent diagnosed with TS and considering the genetic basis for TS, it felt important to capture their mother's experiences. Future research may benefit from exploring differences among those with different types of tic disorders and having a more narrowly defined age range to account for developmental differences. Finally, almost all the children had comorbidities which meant it was difficult to differentiate which mealtime difficulties were strictly related to TS, and which were related to comorbidities. While this is a limitation of the study, it is also representative of a TS sample. This sample is thought to reflect the spectrum of presentations within this population, with TS being a multifaceted condition with a complex clinical presentation due to high comorbidity rates (49, 50). Importantly, anomalous eating patterns have been found in children with TS, even when accounting for comorbidities (2), with mothers in the current study, able to shed further light on how their child's symptoms of TS and associated comorbid conditions intersected to make mealtimes complex.

Despite these limitations, this study contributes unique insights by shining a light on some of the hidden challenges mothers may face. This is an essential first step towards designing studies in the future. The hope is that by highlighting the barriers to harmonious and enjoyable mealtimes, practitioners who work with these families may be able to provide mealtime-specific support. The cumulative effect of dissatisfaction, stress and additional foodwork can have a diminishing effect on maternal and familial resilience and wellbeing. In order to provide ongoing care for children with chronic conditions and their families, more emphasis needs to be placed on barriers to meaningful daily activities such as mealtimes. As such, families may benefit from individualised support that can help them create meaningful experiences, be it adjusted mealtimes to accommodate for their challenges or finding alternative bonding activities.

## Data availability statement

Inquiries regarding the data supporting this article can be directed to the corresponding author.

## Ethics statement

The studies involving human participants were reviewed and approved by Ethical approval for this research was obtained from the University of Hertfordshire University Ethical Advisory Committee Protocol Number: aHSK/PGT/UH/03340(5) and the research was performed in accordance with the Declaration of Helsinki. The patients/participants provided their written informed consent to participate in this study. Written informed consent was obtained from the individual(s) for the publication of any potentially identifiable images or data included in this article.

## Author contributions

S-EB conducted the research as part of her PhD, planned research, recruited participants, collected data, transcribed interviews, conducted analysis, revised themes, wrote up the study as a PhD chapter, and drafted the manuscript. AL and WW were the second supervisors of PhD, supported research design, data analysis, and contributed to the writing of the manuscript. SR was the primary supervisor of the PhD, supported research design, data analysis, and contributed to the writing of the manuscript. SR, WW, and AL contributed equally to the drafting of the manuscript. All authors approved the manuscript for submission.

## Funding

This research was completed as part of a PhD in Food and Public Health funded by the Centre for Research in Public Health and Community Care (46). Some of the authors are part-funded or supported by the National Institute for Health and Care Research (NIHR) Applied Research Collaboration East of England. The University of Hertfordshire covered the open access publication fee.

## Conflict of interest

The authors declare that the research was conducted in the absence of any commercial or financial relationships that could be construed as a potential conflict of interest.

## Publisher's note

All claims expressed in this article are solely those of the authors and do not necessarily represent those of their affiliated organizations, or those of the publisher, the editors and the reviewers. Any product that may be evaluated in this article, or claim that may be made by its manufacturer, is not guaranteed or endorsed by the publisher.

## Author disclaimer

The views expressed are those of the author(s) and not necessarily those of the NIHR or the Department of Health and Social Care.
